# Supplementary figures and images for: Interneuronal network model of theta-nested fast oscillations predicts differential effects of heterogeneity, gap junctions and short term depression for hyperpolarizing versus shunting inhibition
Source: PLoS Comput Biol. 2022 Dec 1;18(12):e1010094. doi: 10.1371/journal.pcbi.1010094 (PMC9747050; doi:10.1371/journal.pcbi.1010094)

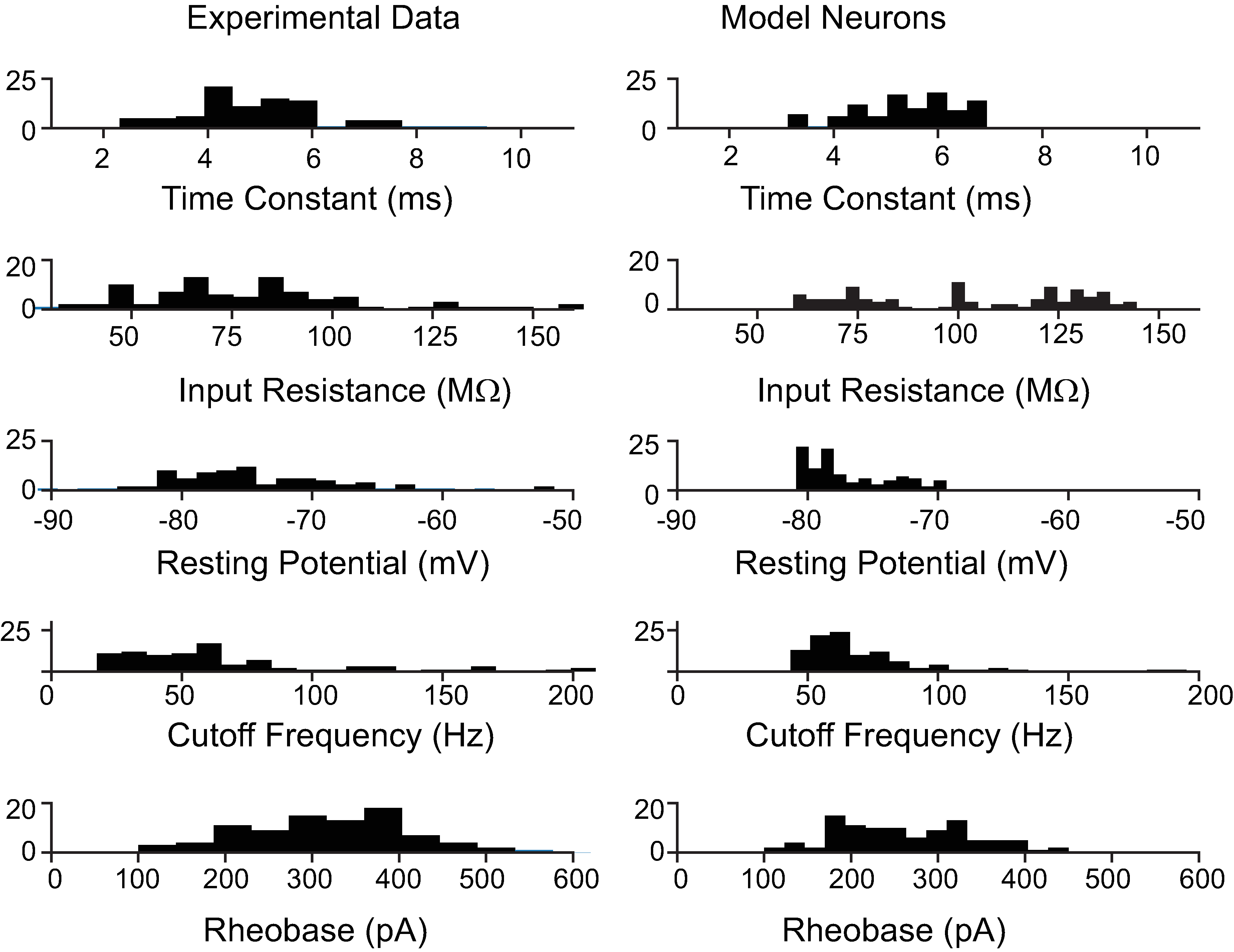

Supplement: S1 Fig — Histograms of passive and active properties of experimental (left) and model neurons (right). A. Time constants. B Input resistance. C Resting Potential. D. Cutoff frequency E. Rheobase. (TIF) [file pcbi.1010094.s001.tif]

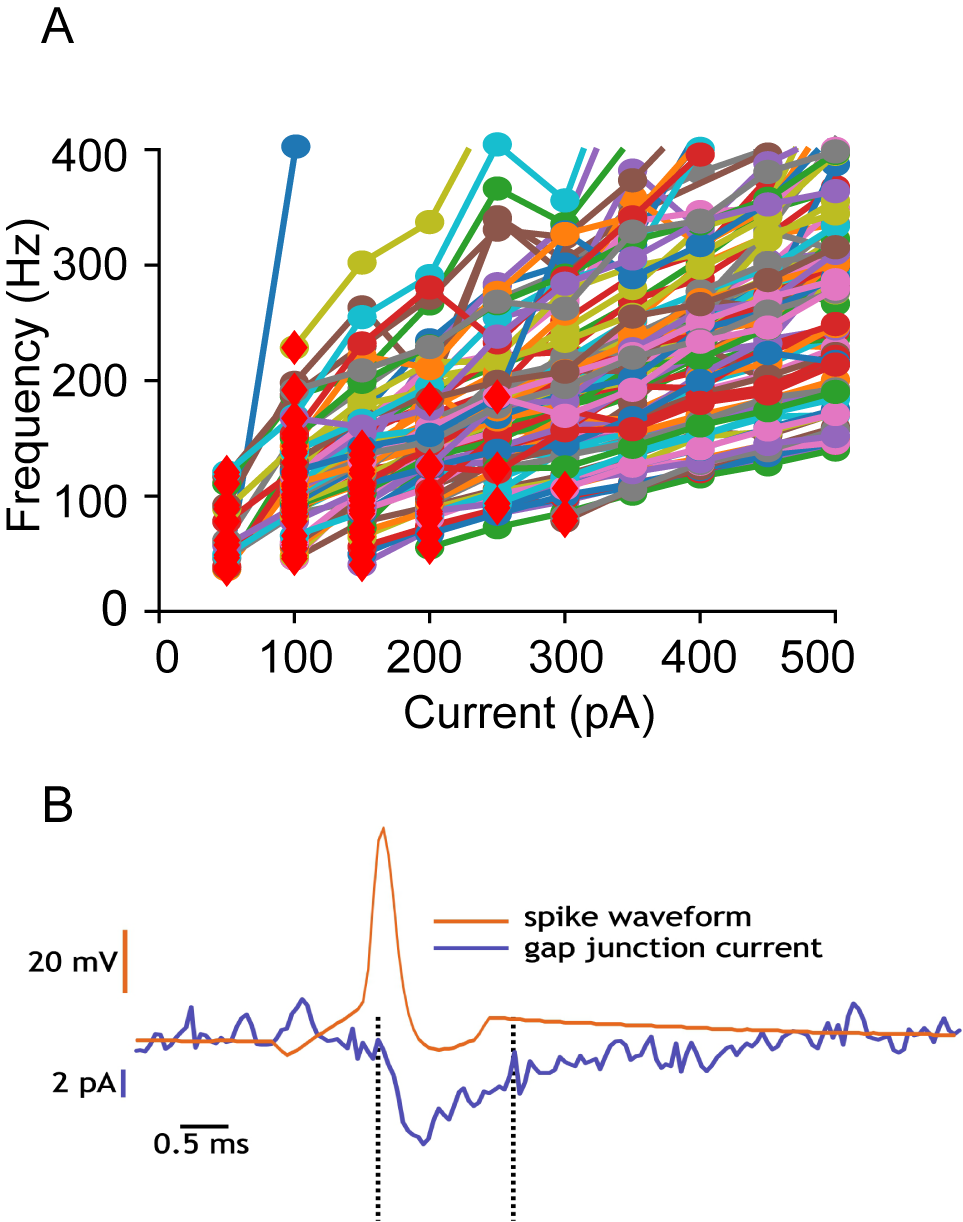

Supplement: S2 Fig — A. Curves for one network instantiation. Adding gap junctions at a few values of injected current on a small number of model neuron f/I curves, possibly due to rebound spiking in another neuron strongly coupled to the injected neuron. B. An example from a pair of PV cells connected by gap junctions showing the gap junction current recorded under voltage clamp at -40 mV in one neuron during a single action potential generated with a brief, large pulse of current (dashed lines) in the other neuron. The bulk of the gap junction current flowed during the downstroke of the spike and the subsequent AHP [42]. (TIF) [file pcbi.1010094.s002.tif]

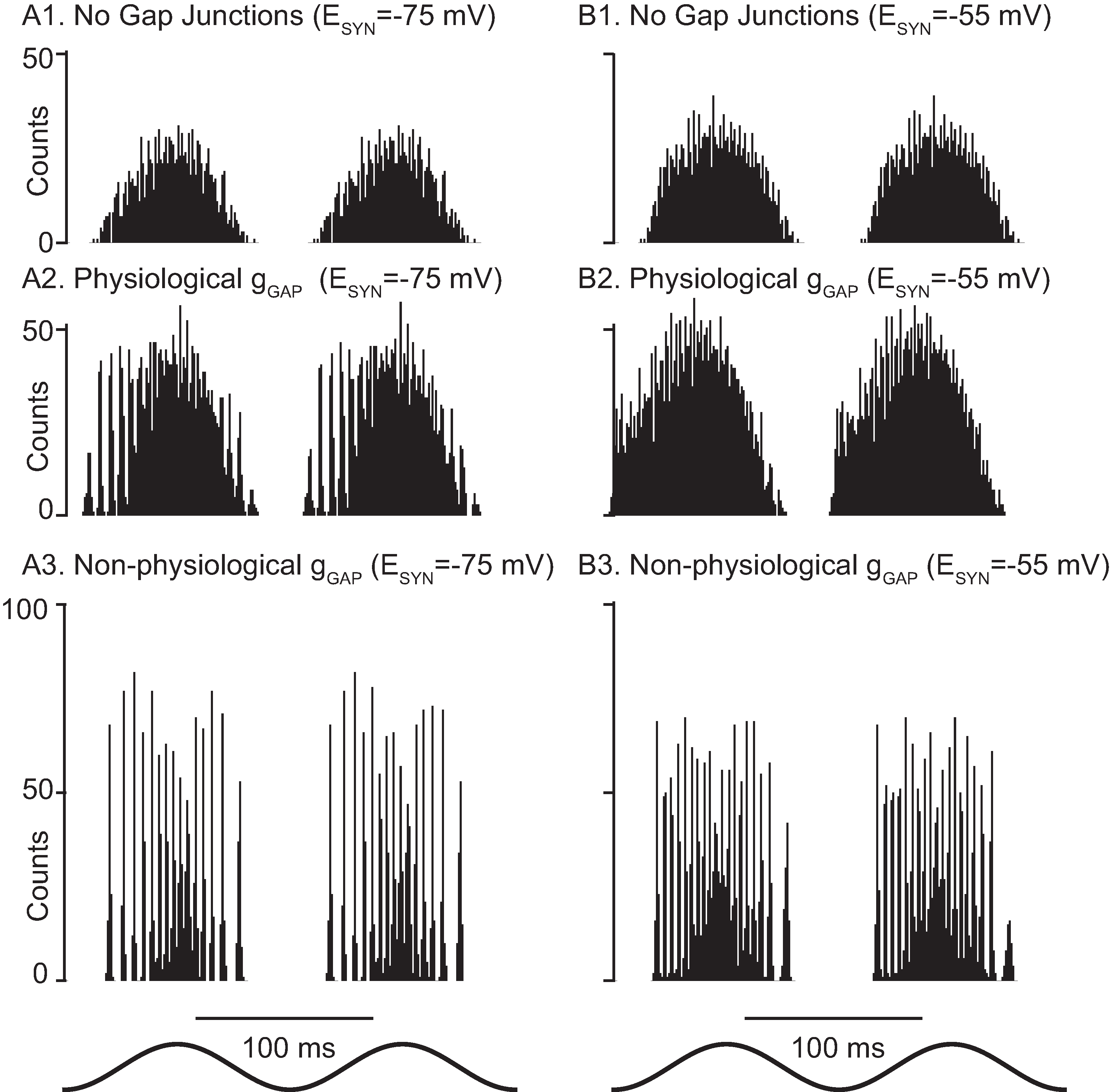

Supplement: S3 Fig — (TIF) [file pcbi.1010094.s003.tif]

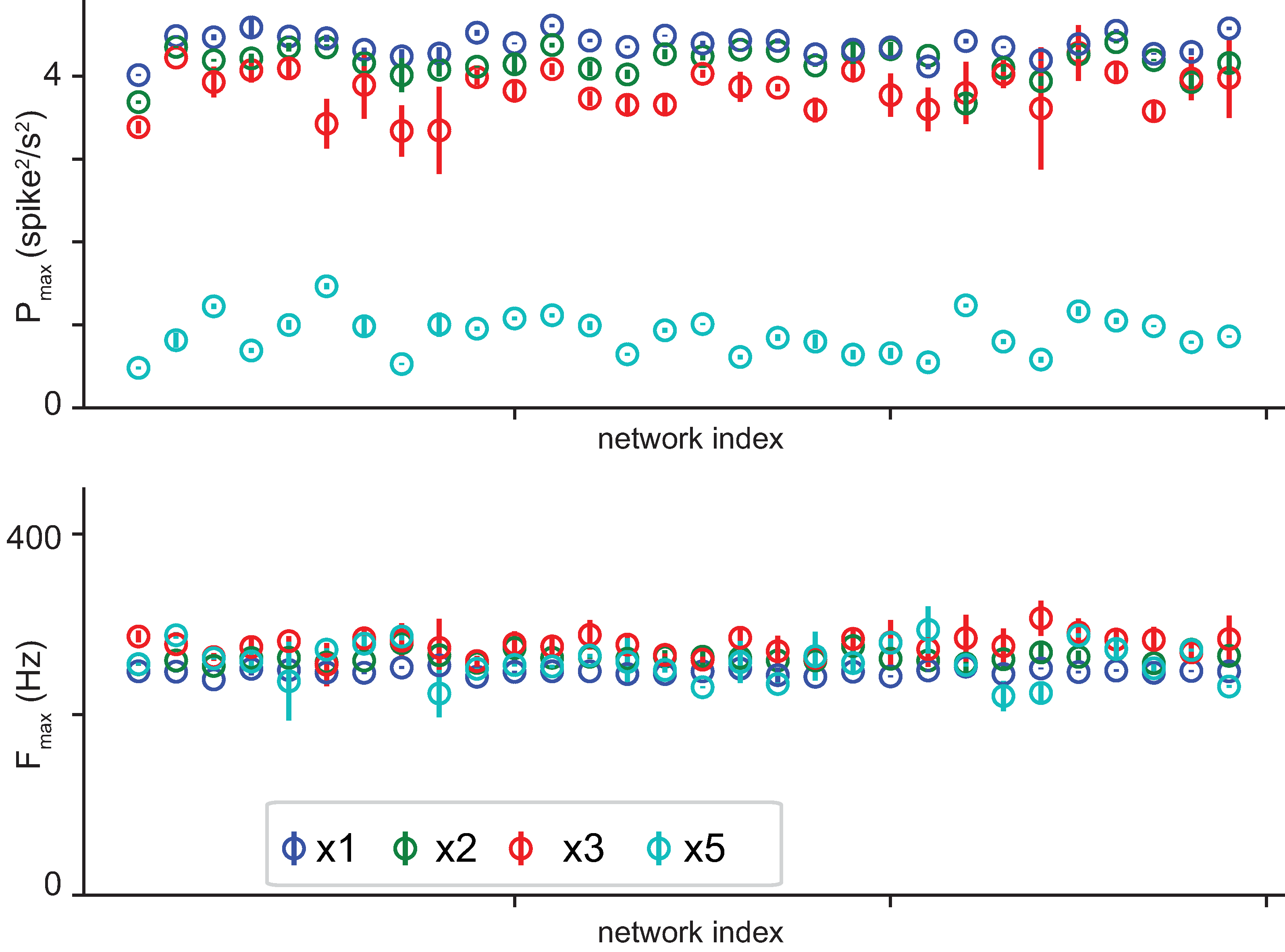

Supplement: S4 Fig — (TIF) [file pcbi.1010094.s004.tif]

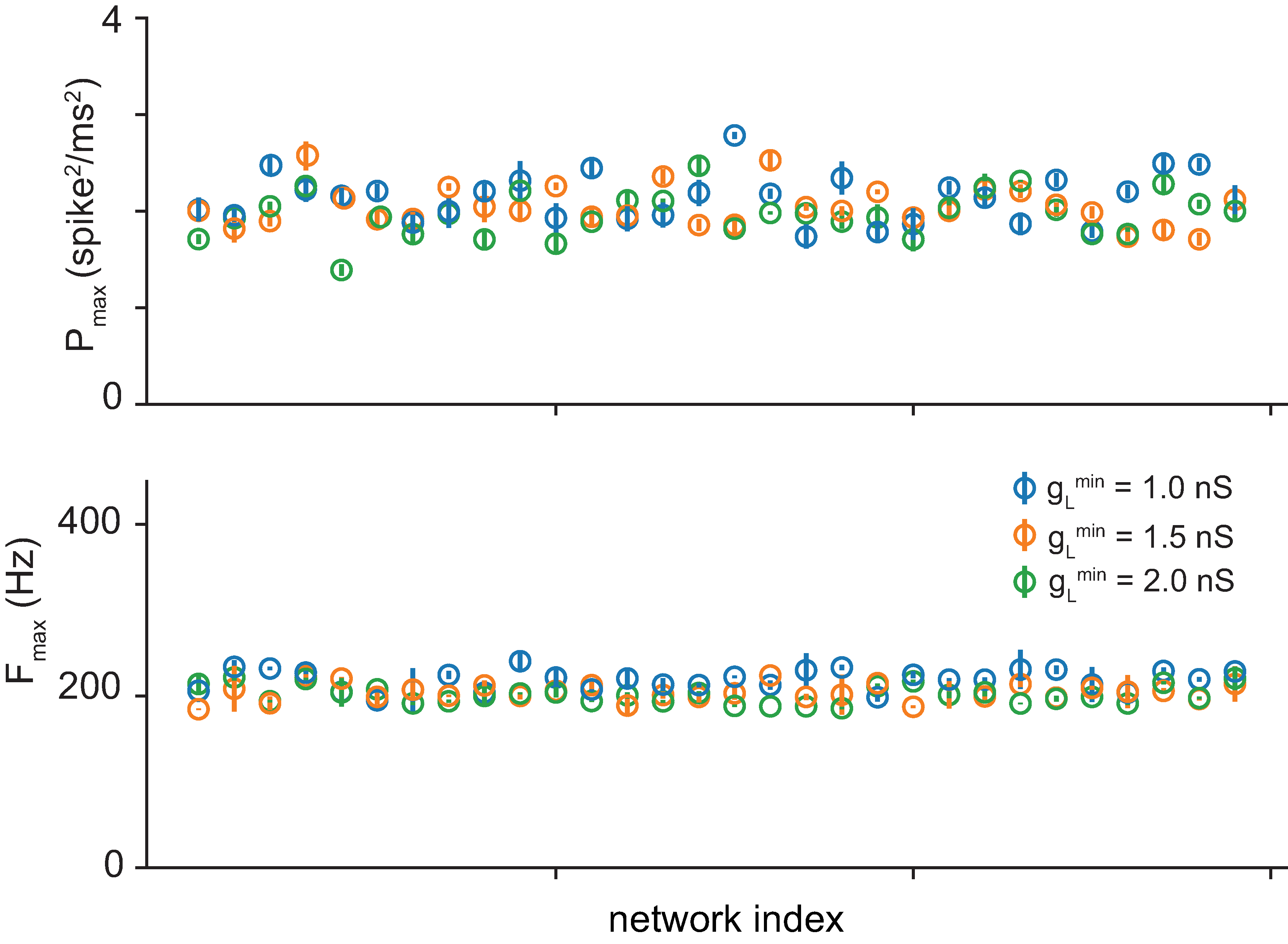

Supplement: S5 Fig — (TIF) [file pcbi.1010094.s005.tif]
