# Supplementary material for: Interneuronal network model of theta-nested fast oscillations predicts differential effects of heterogeneity, gap junctions and short term depression for hyperpolarizing versus shunting inhibition
Source: PLoS Comput Biol. 2022 Dec 1;18(12):e1010094. doi: 10.1371/journal.pcbi.1010094 (PMC9747050; doi:10.1371/journal.pcbi.1010094)
Supplement: Attachment — Submitted filename: Report_PLOS.pdf [file pcbi.1010094.s006.pdf]

The authors develop a detailed model of PV+ interneurons in the medial enthorinal cortex (mEC) including electrical, chemical synapses as well as short-term depression (STD) based on experimental measurements. Their aim is to reproduce theta-nested gamma oscillations at high frequencies (200 Hz) measured via optogenetic techniques in the mEC and associated to an ING mechanism. They show that electrical synapses are required to have synchronization in this ING model due to the level of heterogeneity among PV+ neurons and that this is achieved with biophysical values for hyperpolarizing inhibition and not for shunting inhibition, which has a desynchronizing effect in this context. STD has the role to promote the emergence of fast oscillations before the peak of theta drive, while without STD, they can appear symmetrically before and after the peak. The main novelties of the presented model are : 1) the PV+ model is assumed to be of type 2 excitability; 2) heterogeneity is present both in passive and active parameters.

I find the analysis extremely interesting and the reported results stimulating, however I have a series of remarks and comments that the authors should address before that the manuscript could be considered for publication.

Main remarks :

(M1) The authors seem not to be aware of recent numerical/theoretical work discussing PING and ING generation mechanisms for theta-nested gamma oscillations in terms of a new class of neural mass models for heterogenous spiking neural populations:

[1] M. Segneri, H.Bi, S. Olmi, A.Torcini, "Theta-nested gamma oscillations in next generation neural mass models", *Frontiers in Computational Neuroscience* , 14:47 (2020)

In particular, the work [1] above compares the model findings with recent optogenetic experimental results of Butler group [ref 13 of the authors] and reference [2] below :

[2] Butler, J. L., Mendonça, P. R., Robinson, H. P., and Paulsen, O. (2016). Intrinsic cornu ammonis area 1 theta-nested gamma oscillations induced by optogenetic theta frequency stimulation. J. Neurosci. 36, 4155–4169. doi: 10.1523/JNEUROSCI.3150-15.2016

this work [2] has not been cited by the authors, despite its relevance.

(M2) Another relevant work not cited by the authors, where heterogeneous inhibitory neural networks giving rise to fast and slow gamma oscillations as well as phase locking with the theta drive are considered is the following:

[3] H. Bi, M. Segneri, M. di Volo,A.Torcini, "Coexistence of fast and slow gamma oscillations in one population of inhibitory spiking neurons", *Physical Review Research* ,2, 013042 (2020)

In this work a different source of heterogeneity is considered with respect to [1], the one associated to the random distribution of the connections.

(M3) Other quite relevant experimental results based on optogenetic stimulation not cited by the authors are reported in the following article:

[4] Akam, T., Oren, I., Mantoan, L., Ferenczi, E., and Kullmann, D. M. (2012). Oscillatory dynamics in the hippocampus support dentate gyrus-ca3 coupling. Nat. Neurosci. 15:763. doi: 10.1038/nn.3081

(M4) I do not find particularly informative the mean field analysis based on a homogeneous network, the authors seem not to be aware of recent mean field results based on a new class of neural mass models for heterogeneous spiking networks discussing the relevance of phase response curve for locking in ING and PING mechanism:

[5] Dumont, G., and Gutkin, B. (2019). Macroscopic phase resetting-curves determine oscillatory coherence and signal transfer in inter-coupled neural circuits. PloS Comput. Biol. 15:e1007019. doi: 10.1371/journal.pcbi.1007019

see also :

[6]
Dumont, Grégory, G. Bard Ermentrout, and Boris Gutkin. "Macroscopic phase-resetting curves for spiking neural networks." *Physical Review E* 96.4 (2017): 042311.

(M5) May the authors attempt to introduce some heterogeneity in their mean field model ? This will render the results more meaningful and useful to compare with the network simulations, as done in [1],[5], and [6] cited above, where the heterogeneity was only in the neuron excitabilities or as in [3] where the heterogeneity was in the random distribution of the connections. Furthermore, heterogeneous mean field models for spiking networks with short-term synaptic plasticity have been also recently analyzed in :

[7] H. Taher, A. Torcini, S. Olmi, "Exact neural mass model for synaptic-based working memory", *PLOS Computational Biology* , 16(12): e1008533 (2020)

Maybe the authors can use these mean field models to make predictions on their spiking networks, despite the neurons here considered are of type I excitability

(M6) The evidence that electrical synapses are required to have synchronization in this ING model due to the level of heterogeneity and that this is achieved with biophysical values for hyperpolarizing inhibition and not for shunting inhibition is shown in Fig 6 A2, and Fig 7. May the authors compare their finding with experimental ones in more details ? To show that in mEC indeed we observe a similar behaviour , e.g. that the high synchronization is present only in the first part of the theta cycle. These results seems to depend at least on the frequency of the forcing theta cycle, as shown in Fig 4A in [2] : slow theta favours the appearance of gamma burst on the rising part of the theta cycle; while high theta frequency favours the emergence of the gamma oscillation on the descending part of the theta cycle. May the authors analyze the influence of forcing with different frequencies ?

(M7) I do not understand the difference among the results reported in Fig. 6A2 and Fig. 8A2. For what I have understood both figures refer to the same set-up: inhibitory network in absence of STD for hyperpolarizing synapses with realistic values of the gap junction parameters. If this is not the case please specify in the text. If this is the case please explain why in Fig 8A2 no phase preference is present for gamma oscillations.

(M8) The authors do not address the role of autaptic self-connections in PV inter-neurons, despite these autapses seem to be fundamental for preventing high gamma bursts and favouring locking with gamma oscillation in the usual range, as recently shown in :

[8] Deleuze, Charlotte, et al. "Strong preference for autaptic self-connectivity of neocortical PV interneurons facilitates their tuning to γ-oscillations." *PLoS Biology* 17.9 (2019): e3000419.

May the authors discuss this aspect and which could be the role of autapses in their model ?

(M9) I think the authors should include in their discussion of the results also the recent theoretical and experimental papers I mentioned above.

Minor remarks:

(1) A wavelet analysis analogous to the one reported in Fig. 4 G in reference [2] above will help in understanding the relationship between gamma frequency oscillations and theta phase in the different studied cases ;

(2) It would be also useful to see the frequency response of the model for different values of the theta frequency forcing, as well as of forcing amplitude;

(3) Figure 6 - In the caption the authors refer to panels A,B,C, which are not present in the figure.

(4) The authors mention Fig 7A2 in the caption of Fig 8, I am unable to find this figure;

In summary I find the manuscript well written and reporting new interesting results, apart a few missing citations of recent theoretical and experimental results on theta-gamma oscillations. However, I find that the most critical aspect of the reported analysis concerns the mean field approaches for the PRC. As recently shown in [5,6] macroscopic PRCs for heterogeneous networks can be indeed derived. I think the authors could include the effect of hyperpolarizing or shunting inhibition in such heterogeneous mean field models by following e.g. the derivation reported in

[9] Coombes, Stephen, and Áine Byrne. "Next generation neural mass models." *Nonlinear dynamics in computational neuroscience*. Springer, Cham, 2019. 1-16.

and those due to STD by following the approach in [7].
