# Supplementary material for: Interneuronal network model of theta-nested fast oscillations predicts differential effects of heterogeneity, gap junctions and short term depression for hyperpolarizing versus shunting inhibition
Source: PLoS Comput Biol. 2022 Dec 1;18(12):e1010094. doi: 10.1371/journal.pcbi.1010094 (PMC9747050; doi:10.1371/journal.pcbi.1010094)
Supplement: Attachment — Submitted filename: responsefinal.docx [file pcbi.1010094.s007.docx]

Response to Reviewers

*We thank the reviewers for their constructive criticisms. We have recalibrated the model to incorporate all available data on layer 2/3 MEC PV+ neurons, and remarkably, the major results were all robust to the recalibration. We split the PRC figure into two figures and added panels to Figure 7 to show the effect of varying the amplitude and frequency of the drive. We feel that the paper is much improved due to the constructive feedback provided by the reviewers. Below is a point by point response detailing all changes.*

Reviewer Fig. 1

Reviewer #1:

1. Despite the great articulation of the topic that authors provide, the paper by its own nature has many compounding parts. As a result, it is hard to leave this paper with a unified picture of what one learns from this work. To possibly mitigate this factor, I would like to suggest that, in the spirit of delivering a comprehensive yet succinct message that the reader can ‘take home’, findings could be summarized in some schematic representation that recapitulates the main roles that each factor plays in influencing network dynamics. I am referring to a pictorial representation, not actual simulations or data, since those are appropriately proposed in the collection of figures shared with the reader.

*The main ideas are that gap junctions, hyperpolarizing synapses with some delay, and depression of shunting synapses promote synchrony, whereas heterogeneity, depression of hyperpolarizing synapses, and shunting synapses oppose it. We added the figure to the left as Fig. 9 in the discussion to summarize the results. It is not a schematic, but it was the simplest way we could think of to depict the major points pictorially.*

2. Line 179 (Figure 4). The authors state: “the perfect synchrony present with chemical synapses intact indicates that the synapses themselves are not sufficient to destabilize global synchrony in the presence of a common sinusoidal drive”. Does that imply that you have checked and the synchronous state you see with synapses present is the same as the synchronous state you will get without any synapse? Or, while one finds global coordination in both cases, the synapses do introduce some difference in the rhythm? I am thinking of mean fields and how to assess the ‘field response’ to input one would consider both the intrinsic properties of the cell population (the f-I curves, for example) and the synaptic currents within the network to establish the regional response to input. So, I am guessing that synapses still do something, possibly even something that promotes stability, rather than de-stabilize? I feel this question is partly answered later in the paper, but for a reader it arises at this point, so it should be at least partially addressed here.

*The shunting synapses in Fig. 3B1 (formerly Fig. 4B1) are weakly desynchronizing, and Fig S4 directly shows that increasing the shunting conductance fivefold leads to less synchrony in the presence of the unphysiologically strong gap junctions required to synchronize our shunting networks. However, to address the reviewer’s valid point, we added the following sentence in lines 166-168:*

*“*Removing synaptic connectivity in a sinusoidally-driven homogeneous network only alters the timing of the population spikes, and not their synchrony (not shown)*.”*

*We include the simulations of Fig. 3A1 and B1 with chemical synapses set to zero for comparison here:*

Reviewer Fig. 2 showing theta drive synchronizes unconnected network.

3. Regarding a step in the methodology to fit model parameters to experimental properties. What is the rationale for choosing uniform sampling (as opposed to other types, say Gaussian) in a network of 100 neurons? Were the values found among the real cells uniformly distributed?

*We did not describe the model calibration in sufficient detail. In response to this point and Reviewer 3’s point 3 below, we have completely recalibrated the model. The distribution of all parameters, experimental and modeled, are now given in Supplemental Figure 1. The recalibration did not alter the main results. We added this description to the text in lines 101-132 describing Fig. 1 (formerly Fig. 2):*

“*Our objective was to capture the heterogeneity in the interneuronal population as faith-fully as possible. Whereas many previous studies kept the f/I curve for the neurons constant and simply varied rheobase by manipulating the bias current, we emphasized fitting the envelopes of the f/I curves across the population. Step currents were applied to determine whether physiological neurons (Fig. 1A1) or model neurons (Fig. 1A2) could support repetitive firing at various levels of current as shown in Fig. 1B. The stabilized steady frequency was recorded (see Methods) and plotted as the f/I curves (Fig. 1B). While maintaining the approximate distribution of cutoff frequencies (the minimum frequencies below which repetitive firing could not be sustained) and rheobases, we also attempted to simultaneously fit multiple additional constraints, described below, that ensured the voltage traces observed during simulated measurement of the f/I curves (Fig. 1A2) were similar to the experimentally observed ones (Fig. 1A1), using only values of passive properties in the measured physiological range. The resting potential, time constant and input resistance ranges are consistent with an earlier study* (Martínez et al. 2017) *and given in the Methods. The AP amplitude (~40 mV above threshold), half-width (~0.3 ms) and AHP depth (~20 mV) were constrained within the range of experimentally observed values. The parameters for the Na and Kv3 currents were calibrated (see Methods) in the absence of Kv1 to reproduce the action potential waveform. Moreover, in some model neurons accumulation of Na inactivation and Kv1 activation were calibrated to exhibit the weak early spike frequency adaptation observed in some recorded neurons, with the additional detail that in some model neurons K_V_1 parameters were calibrated to emit one or more spikes then fall silent at values of injected current too weak to sustain repetitive firing (Fig. 1B). Our results are consistent with previous studies showing that Kv1 and Kv3 set the minimum and maximum firing rates, respectively* (Goldberg et al. 2008; Gu et al. 2018)*. Applying all of these constraints to the selection of model neurons did not allow us to honor the shape of the distributions of all measured passive properties, rheobase and cutoff frequency exactly (Supplementary Figure 1A-E); however, all model values are within the experimentally observed ranges*.*”*

*Please also see the response to Reviewer 3, point 3, which gives more details on the calibration.*

4. Line 186: “However, the peaks widen and diminish in height until fast oscillations disappear. The synchronization properties of the same network using shunting chemical synapses also decays but not as abruptly.” Did you look at these time scales? Is this effectively a transient gamma inside some theta phases? Again, I feel this is partly addressed later, but the reader encounters the question at this stage. Perhaps just a brief sentence to acquiesce curiosity at this stage is all that’s needed.

*The recalibration altered these results a bit, so we replaced those sentences with this one in lines 176-177:*

*“However, near perfect global synchrony persists only for the first few population spikes.”*

*We added this sentence at the end of the paragraph in lines 177-179:*

*“The mechanisms underlying the decrease in synchrony during the theta cycle will be investigated first with respect to the phase resetting properties of the two types of syn-apses and subsequently with respect to the effects of synaptic depression.”*

5. Figure 5. I enjoyed the result section pertinent to figure 5 and appreciate the effort put in delivering intuitive understanding of PRC and their usefulness to a broader audience. However, I would encourage the authors to consider splitting figure 5A in two figures, one for the shunting network and one for the hyperpolarizing current network. This is because it would strongly simplify following along the text from the figure, which currently has too many facts crowded together. As a result, one ‘sees’ all these facts as once when referred to the figure, but only gets to understand what all those curves and arrows and ranges are doing much later in the text.

*Done. We separated the original Fig. 5 into a new Fig. 4 for hyperpolarizing inhibition and a new Fig. 5 for shunting inhibition.*

6. A few small things:

A: Typo line 518 pars “reticulate” should be pars reticulate

*Typo corrected.*

B: Typo in line 105, a comma inside the parenthesis

*Typo corrected.*

C: You mean figure 2C1 and 2C3 (not B) in line 143 (I think)

*That line was deleted due to the recalibration.*

Reviewer #2:

(M1) The authors seem not to be aware of recent numerical/theoretical work discussing PING and ING generation mechanisms for theta-nested gamma oscillations in terms of a new class of neural mass models for heterogenous spiking neural populations:

[1] M. Segneri, H.Bi, S. Olmi, A.Torcini, "Theta-nested gamma oscillations in next generation neural mass models", Frontiers in Computational Neuroscience , 14:47 (2020)

In particular, the work [1] above compares the model findings with recent optogenetic experimental results of Butler group [ref 13 of the authors] and reference [2] below :

[2] Butler, J. L., Mendonça, P. R., Robinson, H. P., and Paulsen, O. (2016). Intrinsic cornu ammonis area 1 theta-nested gamma oscillations induced by optogenetic theta frequency stimulation. J. Neurosci. 36, 4155–4169. doi: 10.1523/JNEUROSCI.3150-15.2016

this work [2] has not been cited by the authors, despite its relevance.

*We tried to focus specifically on inhibitory rhythms in entorhinal cortex, hence we cited (Butler et al. 2018) but not optogenetically induced gamma in other regions like* (Butler et al. 2016) *on PING in CA1. However, we now cite [1] several times, for example in lines 600-604, see response to M9 below.*

*“One very interesting finding from* (Segneri et al. 2020) *is that a theta phase preference, opposite to the one created by synaptic depression in our study, was observed as the theta forcing drives the neural mass model through a subcritical Hopf, causing a bias for nested gamma towards later rather than early theta phases.”*

(M2) Another relevant work not cited by the authors, where heterogeneous inhibitory neural networks giving rise to fast and slow gamma oscillations as well as phase locking with the theta drive are considered is the following:

[3] H. Bi, M. Segneri, M. di Volo,A.Torcini, "Coexistence of fast and slow gamma oscillations in one population of inhibitory spiking neurons", Physical Review Research ,2, 013042 (2020)

In this work a different source of heterogeneity is considered with respect to [1], the one associated to the random distribution of the connections.

*We now cite [3], see response to M9 below.*

(M3) Other quite relevant experimental results based on optogenetic stimulation not cited by the authors are reported in the following article:

[4] Akam, T., Oren, I., Mantoan, L., FerencziE., and Kullmann, D. M. (2012). Oscillatory dynamics in the hippocampus support dentate gyrus-ca3 coupling. Nat. Neurosci. 15:763. doi: 10.1038/nn.3081

*We agree that the work by Akam et al is important but prefer to focus on MEC as stated in the response to M1.*

(M4) I do not find particularly informative the mean field analysis based on a homogeneous network, the authors seem not to be aware of recent mean field results based on a new class of neural mass models for heterogeneous spiking networks discussing the relevance of phase response curve for locking in ING and PING mechanism:

[5] Dumont, G., and Gutkin, B. (2019). Macroscopic phase resetting-curves determine oscillatory coherence and signal transfer in inter-coupled neural circuits. PloS Comput. Biol. 15:e1007019. doi: 10.1371/journal.pcbi.1007019

see also :

[6] Dumont, Grégory, G. Bard Ermentrout, and Boris Gutkin. "Macroscopic phase-resetting curves for spiking neural networks." Physical Review E 96.4 (2017): 042311.

*We now include an entire discussion section on neural mass models in lines 596-642 and cite both of these papers, see response to M9. In that paragraph we also attempt to clarify the motivation behind our mean field analysis and the insights that it provides.*

(M5) May the authors attempt to introduce some heterogeneity in their mean field model? This will render the results more meaningful and useful to compare with the network simulations, as done in [1],[5], and [6] cited above, where the heterogeneity was only in the neuron excitabilities or as in [3] where the heterogeneity was in the random distribution of the connections. Furthermore, heterogeneous mean field models for spiking networks with short-term synaptic plasticity have been also recently analyzed in :

[7] H. Taher, A. Torcini, S. Olmi, "Exact neural mass model for synaptic-based working memory", PLOS Computational Biology , 16(12): e1008533 (2020)

Maybe the authors can use these mean field models to make predictions on their spiking networks, despite the neurons here considered are of type I excitability

*The mass models referred to in general assume current based synapses, such that the inputs can be linearly summed. In addition, they assume that the very nature of the heterogeneity in “excitability” can be captured by a Lorentzian distribution in what is essentially a bias current that is different for otherwise identical neurons. Use of the Lorentzian distribution is simply a mathematical convenience, as similar results have been observed for Gaussian distributions.*

*With respect to the mean field model, we apologize for not making it clear that the phase response curve we use are not infinitesimal and that the response to simultaneous inputs cannot be linearly summed. Instead we use what others have termed a spike response curve. This is now clarified in the description of Figure 4, see lines 197-199 and 199-203.:*

*“In order to apply phase response theory under the assumption of pulsatile coupling, an autonomous system with constant parameters is required. …We generated a phase response curve (sometimes called a spike response curve [30,31]) by applying an inhibitory postsynaptic conductance on separate trials at each of 100 equally spaced phases within the free-running cycle of the model neuron”*

*The shape and magnitude of the PRC under assumptions of strong pulsatile coupling is highly dependent on the conductance values of the perturbations, the effect of 36 synchronous inputs is not 36 times the effect of a single input. Also, if the inputs are not received simultaneously, there is no good way to account for the spread because the neurons have not returned to their limit cycle between the times two inputs are received, so both the phase and the phase resetting are undefined. The only case for a large network that we know how to treat exactly is a homogeneous system in which large numbers of neurons can be assumed to fire simultaneously. See also the response to M9 below.*

(M6) The evidence that electrical synapses are required to have synchronization in this ING model due to the level of heterogeneity and that this is achieved with biophysical values for hyperpolarizing inhibition and not for shunting inhibition is shown in Fig 6 A2, and Fig 7. May the authors compare their finding with experimental ones in more details ? To show that in mEC indeed we observe a similar behaviour, e.g. that the high synchronization is present only in the first part of the theta cycle. These results seems to depend at least on the frequency of the forcing theta cycle, as shown in Fig 4A in [2] : slow theta favours the appearance of gamma burst on the rising part of the theta cycle; while high theta frequency favours the emergence of the gamma oscillation on the descending part of the theta cycle. May the authors analyze the influence of forcing with different frequencies ?

*Experimental work is ongoing with respect to showing that a preference for the first part of the theta cycle is observed in optogenetically driven theta-nested gamma; the results are promising but not yet ready for publication.*

*The suggestion to look at the effect of different forcing frequencies is well-taken, see response to minor point 2 below. We added two panels to Fig. 7 exploring the effects of varying the amplitude at two frequencies, see text in lines 396-410 :*

“Next we examined the effect of varying the amplitude of the theta drive at the same frequency as in the previous panel (Fig. 7B) and at a slower frequency, 4 Hz (Fig. 7C). The frequency at which the maximum power was observed saturated at about 200 Hz for both driving frequencies, and the maximum power saturated as well. During the rising phase of the theta drive, if the amplitude of drive reaches a level that allows a network frequency of about 200 Hz to be attained, the network desynchronizes as described in the next paragraph. The frequency with maximum wavelet power occurs just prior to desynchronization. Many manipulations may decrease (increase) the frequencies relative to a fixed theta drive, such as increasing (decreasing) the synaptic decay time, increasing (decreasing) the time constant of individual neurons, or slowing (speeding) the deactivation of K_V_3. However, the maximum network frequency, followed by desynchronization, is simply reached earlier or later within the theta cycle. In the absence of STD, slightly higher frequencies can be achieved (250 Hz) before the network desynchronizes; the inhibition from successive population spikes summates at high frequencies and becomes more tonic and less phasic (White et al. 1998), likely favoring desynchronization.”

(M7) I do not understand the difference among the results reported in Fig. 6A2 and Fig. 8A2. For what I have understood both figures refer to the same set-up: inhibitory network in absence of STD for hyperpolarizing synapses with realistic values of the gap junction parameters. If this is not the case please specify in the text. If this is the case please explain why in Fig 8A2 no phase preference is present for gamma oscillations.

*We apologize for lack of clarity, the figure caption for Figure 6 was wrong and has been corrected. In Fig. 6A2 synaptic depression is included, whereas in Fig. 8A2 synaptic depression is not present. Since the phase preference is due to a decrease in synchrony as hyperpolarizing inhibition is depressed, the absence of depression in Fig. 8A2accounts for the lack of phase preference.*

(M8) The authors do not address the role of autaptic self-connections in PV inter-neurons, despite these autapses seem to be fundamental for preventing high gamma bursts and favouring locking with gamma oscillation in the usual range, as recently shown in :

[8] Deleuze, Charlotte, et al. "Strong preference for autaptic self-connectivity of neocortical PV interneurons facilitates their tuning to γ-oscillations." PLoS Biology 17.9 (2019): e3000419.

May the authors discuss this aspect and which could be the role of autapses in their model ?

*Reviewer Figure3 (paper figure 3 with autapses)*

*We thank the reviewer for the (Deleuze et al. 2019) citation. That study examined autapses in neocortex, and the extent of the contribution of autapses to inhibition onto PV+ cells in the MEC is unknown. However, per the reviewer’s suggestion, we did examine the effects of autapses*. *To account for autapses, we redid the simulations under the assumption that about a third of the inhibition was self-inhibition. For a fair comparison, we kept the total amount of synapses the same, so we reduced the network contribution to 24 synapses and added an autapse that was 12 times as strong as an individual synapse. Adding the hyperpolarizing autapses caused the free running period of the single neuron to increase and adding shunting decreased the period, but the network results were quite similar (see for example Reviewer Figure 3). We added a sentence to the paper in lines 318-321 stating that*

*“Recent work in neocortex found that a significant fraction of the inhibition received by PV+ interneurons in that area was mediated by autapses (Deleuze et al. 2019).The synchronization properties of both hyperpolarizing and shunting networks were unchanged by shifting a third of the inhibition from synapses from other interneurons to autapses.*”

(M9) I think the authors should include in their discussion of the results also the recent theoretical and experimental papers I mentioned above.

*We now include the following paragraphs in a new Discussion section:*

*Neural Mass Models and the Mean Field Approach*

*“Recently, elegant, low dimensional neural mass models have been described that accurately capture the fluctuation in rate during nested gamma of networks of QIF driven at theta* (Segneri et al. 2020) *and capture some aspects of optogenetically-driven theta nested gamma in hippocampal area CA1* (Butler et al. 2016)*. One very interesting finding from* (Segneri et al. 2020) *is that a theta phase preference, opposite to the one created by synaptic depression in our study, was observed as the theta forcing drive the neural mass model through a subcritical Hopf, causing a bias for nested gamma towards later rather than early theta phases.” These models are based on some simplifying assumptions. First, the dynamics of the component neurons can be captured by assuming they are one-dimensional integrators of current. Second, the inputs presented to them are current inputs that summate linearly Third, the heterogeneity in the network is captured by giving neurons different bias currents. Fourth, the connectivity is all to all. Fifth, synaptic plasticity is ignored. The last two constraints have been respectively relaxed in recent neural mass models* (Bi et al. 2020; Taher et al. 2020)*, but the first three remain intractable to mass models. In our model, the component neurons are resonators* (Izhikevich 2001) *with Hodgkin’s type 2 excitability* (Hodgkin 1948) *rather than integrators with type 1 excitability* (Izhikevich 2007)*. The chemical and electric synaptic inputs and the external theta drive are not currents, but rather are conductance based. Conductance based inputs do not sum linearly and will saturate. The inclusion of conductance based synapses in neural mass models* (Coombes and Byrne 2019) *on the premise that the neuron is generally at or near its rest potential, thus making the current proportional to the synaptic reversal potential, still assumes that conductances add linearly. In the neural mass models, the different bias currents ensure that the same external input will cause different neurons to be biased at different points along their identical f/I curves. However, the heterogeneity we described here is far more complex and desynchronizing than simple differences in bias current in identical neurons. Our networks contain heterogeneity in the intrinsic passive, intrinsic active and synaptic conductances and connectivity as well as in the kinetics of the active currents.*

*Another approach to neural mass models is to use a pulse-coupled phase oscillator model* (Winfree 1967) *in which each neuron is represented by a phase model. The phase advances at a constant rate determined by the intrinsic frequency when the neuron is unperturbed, but inputs from other neurons advance or delay the phase. A neural mass model based on this strategy* (Dumont et al. 2017; Dumont and Gutkin 2019; Pazó and Montbrió 2014) *cannot be applied to our networks because they assume an infinitesimal PRC of constant sinusoidal shape. This is not consistent with conductance-based synapses in which the shape of the PRC depends on the conductance strength* (Achuthan and Canavier 2009)*. Moreover, for non-instantaneous synapses, the PRC is undefined in the case of an input received before the effect of the previous input has dissipated because the phase is only defined on the limit cycle.*

*Our mean field approach in Figures 4 and 5, is intended to provide insight into the types of manipulations that affect neural systems whose dynamics cannot be known in sufficient detail to model precisely. The only case for a large network that we know how to treat exactly is a homogeneous system in which all neurons can be assumed to fire simultaneously. The pulse-coupled PRC analysis is a vast simplification of the model network, which in turn is a simplification of the experimental preparation. However, it does explain why conduction delays are required for synchrony for strong inhibition* (Tikidji-Hamburyan et al. 2019)*. It also suggests that the steepness of the PRC contributes to the more highly synchronizing effects of hyperpolarizing versus shunting inhibition.”*

(M10). In summary I find the manuscript well written and reporting new interesting results, apart a few missing citations of recent theoretical and experimental results on theta-gamma oscillations. However, I find that the most critical aspect of the reported analysis concerns the mean field approaches for the PRC. As recently shown in [5,6] macroscopic PRCs for heterogeneous networks can be indeed derived. I think the authors could include the effect of hyperpolarizing or shunting inhibition in such heterogeneous mean field models by following e.g. the derivation reported in

[9] Coombes, Stephen, and Áine Byrne. "Next generation neural mass models." Nonlinear dynamics in computational neuroscience. Springer, Cham, 2019. 1-16.

and those due to STD by following the approach in [7].

*These are excellent suggestions but we think they would require a separate, companion paper. We now cite [9], see response to M9 above.*

Minor remarks:

(1) A wavelet analysis analogous to the one reported in Fig. 4 G in reference [2] above will help in understanding the relationship between gamma frequency oscillations and theta phase in the different studied cases ;

*We thank the reviewer for this suggestion. In Figure 8C we now plot the wavelet phase in addition to the power to make the nesting of the high frequency oscillations within theta clearer.*

(2) It would be also useful to see the frequency response of the model for different values of the theta frequency forcing, as well as of forcing amplitude;

*In response to this comment and point 6 above, additional simulations probed the effect of varying forcing frequency and amplitude. We added panels B and C to Figure 7 to explore the effect of varying the amplitude of the drive at two frequencies, 8 Hz and 4 Hz, revealing that the nested oscillatory frequency maximum saturates at around 200 Hz, and that a minimum amplitude of drive is required to reveal a theta phase preference, see figure below with 6 nS rather than 14 nS as the maximum value of the optogenic drive.*

*Reviewer Figure 4 (paper figure 6 with max conductance for gChR= 6 nS)*

(3) Figure 6 - In the caption the authors refer to panels A,B,C, which are not present in the figure.

*Typo corrected, see response to M7 above.*

(4) The authors mention Fig 7A2 in the caption of Fig 8, I am unable to find this figure;

*Typo corrected.*

Reviewer #3:

1. The model considers only the interconnected interneuron network, but excludes principal cells. This is a valid approach to understand the mutually interconnected "pacemaker" interneuron network, but raises the question if interneurons can entrain the activity of the pyramidal cells at the same high frequencies, given the differences in the IN-IN and IN-PC synaptic connections.

*This is an excellent question that we are currently addressing but prefer to include in our next manuscript. We revised the following sentence in lines 535-538 of the Discussion:*

*“Our study focused on ING and did not include excitatory neurons; perhaps in vivo neural populations such as the stellate, pyramidal or other inhibitory neurons contribute to slowing the oscillations into a fast gamma range via synaptic or modulatory mechanisms”.*

2. Basic information on the electrophysiological recordings is completely missing from the ms. Only a reference is made to the previous publication from which data was partially taken. Please provide relevant information in the Methods, including how many new recordings were made for this study and how neurons/ from the previous study were selected for this one.

*Sections on “Transgenic mice and slice preparation” and “Electrophysiology” were added to lines 702-735 of the Methods section. No new recordings were made for this study. We now state in lines 737 and 738 of the Methods that*

*“Methods were as in* (Fernandez et al. 2022)*], with all data taken from recordings collected in that study, and summarized above.”*

3. As the aim here was to calibrate heterogeneity, it is surprising that only 11 interneurons were evaluated in contrast to the 122 pairs recorded on Ref 32. If data was taken form the previous publication, why wasn't the whole data taken to get the best possible estimate of heterogeneity?

*We thank the reviewer, we took this suggestion and recalibrated the 100 neuron network using the entire experimental database (instead of a subset) in terms of the f/I curves and passive properties. This required us to regenerate all figures. The essential results were unchanged, but we now better document both the experimental data and how it was incorporated into the model. Please see response to Reviewer 1 point 3 for changes made to the text describing Figure 1 (formerly Figure 2) as well as the text below which was added in lines 783 to 800 of the Methods.*

“*In order to calibrate the network as described in the text accompanying Figure 1, we generated 100 vectors of length three, each with the three passive parameters: membrane time constant, input resistance and leakage reversal potential. The values for these parameters were drawn from random variables with uniform distribution in ranges similar to the ones observed in the recorded neurons, i.e. time constant from 3 to 7 ms, input resistance from 50 to 150 MOhm, and leakage reversal potential from -80 to -60 mV. The time constant and input resistance were used to set the membrane capacitance: C_m_=τ_m_/R_input_. Some of the parameters (see Table 1) for the voltage-gated currents were uniform across the 100 simulated neurons, but seven active parameters were varied. We generated another set of 100 vectors of length 7 from uniform distributions for the peak conductances for the three voltage-gated ion channels, Nav (g_Na_ 6000 to 35000 nS), Kv1 (g_Kv1_ 15 to150 nS) and Kv3/Na_V_ ratio (g_Kv3_/g_Na_ 0.03 to 0.05) and the activation mid-potentials for the four gating variables, activation and inactivation of Nav (θ_m_ -60 to -45 mV, θ_h_ -60 to -50 mV), and activation of Kv1 (θ_a_ 35 to 55 mV), and Kv3 (θ_n_ -15 to 25 mV). Constraining the Kv3/NaV ratio helped reproduce some of the features from the experimentally recorded traces. Combining each of the 100 passive sets with each of the active sets produced 10,000 parameter sets, from which we selected 100 neurons as described in the text for Fig. 1*.”

4. Finally, it would be also helpful to the reader if the most important aspects (e.g. whole-cell recording in slice preparation, species, mEC layer) would be included in one or two sentences.

*This sentence has been added to the text in lines 72-74 of the Introduction.*

*“We previously performed dual intracellular patch recordings from layer 2/3 mEC in male and female mice expressing the tdTomato fluorophore in PV+ cells* (Fernandez et al. 2022)*, and further analyzed that data in this study”*

5. The frequency range of the oscillations investigated remains poorly defined. While the Abstract and Introduction focus on gamma oscillations, in the Discussion only ripple oscillations are mentioned. While this has no relevance for the results and conclusions, an inconsistent presentation will confuse the readers.

*We revised the following section in lines 521-543 of the Discussion to form a better bridge between fast gamma and ripples:*

*“Fast Oscillations in the Entorhinal Cortex*

*At least two types of fast oscillations have been observed in the entorhinal cortex: fast gamma oscillations in the 65-140 Hz range* (Colgin et al. 2009) *and ripples 140-200 Hz* (Yamamoto and Tonegawa 2017); *they can coexist in vitro when induced by kainate application* (Cunningham et al. 2004)*. The frequency border between these oscillations is ambiguous in the literature; some quote 100 Hz as the border* (Geisler et al. 2005)*, whereas others label 90-150 Hz as an epsilon band or alternatively refer to the 65-90 Hz range as medium gamma and 90-140 Hz as fast gamma* (Belluscio et al. 2012)*. However, gamma oscillations are frequently nested in theta oscillations* (Bragin et al. 1995)*, whereas ripples are often nested in sharp waves* (Chrobak and Buzsáki 1996)*. The frequencies observed in our carefully calibrated model of layer 2/3 mEC PV+ interneuronal networks consistently fall in the upper end of that range (~150-200 Hz). This value is consistent with the frequency of fast oscillations evoked in a study that selectively activated PV+ neurons at theta frequencies* (Williams et al. 2021)*. It is possible that the high frequency oscillations observed in that study and in our model are more analogous to the ripples in superficial mEC that contribute to ripple bursts and extended replays in area CA1 in quiet awake rodents* (Yamamoto and Tonegawa 2017)*. Our study focused on ING and did not include excitatory neurons; perhaps in vivo neural populations such as the stellate, pyramidal or other inhibitory neurons contribute to slowing the oscillations into a fast gamma range via synaptic or modulatory mechanisms. It has been previously suggested* (Mann and Paulsen 2007) *that fast gamma oscillations are more similar to ripples than to slow gamma, and result from interneurons escaping control of phasic excitation and entering a regime of tonic excitation. In the former, the interneurons do not fire unless prompted by the phasic excitation, hence their intrinsic dynamics only contribute to setting the frequency in the tonic excitation regime.*

6. An indirectly related issue is the value of the membrane time constant - the mean value is 5 ms (range 3 - 7 ms), which is approximately twice as fast than other estimates for hippocampal, but also EC basket cells (~10 ms) and also almost 2 ms faster than the mean value in Ref 32 (6.8 ms). This difference might be due to the large current (100 pA) applied to produce the voltage responses for the calculations - with these large currents it is likely that voltage-gated conductances are also activated.

What would be the effect of a slower time constant on the results and conclusions?

*Fernandez et al. measured the time constant in the following way: “a current pulse was used to depolarized cells to a value slightly below spike threshold. A single exponential decay function was used to fit the membrane voltage time course associated with hyperpolarization and the return to resting voltage resulting from the end of the current pulse.” This was done for consistency with the procedure designed to distinguish pyramidal cells from stellate cells in the study, On the other hand, in the current study, “A single rising exponential function was used to fit the voltage trace during the 80 ms following the removal of the -100 pA applied hyperpolarizing current step and the return to the resting membrane potential.” The two different protocols consistently give lower values of the time constant for the latter protocol, so we will keep the lower values. However, additional simulations were run with all time constants increased by 25%. This increased the rheobase and shifts the f/I curves to the right, but does not otherwise substantially affect the results, the frequency of the oscillation was unchanged, compare figure below to Fig. 7A in the manuscript.*

*Reviewer Figure 5 (paper figure 7A with all time constants scaled up by 25%*

7. Synaptic delays were set between 0.6 -1.0 ms, but it is not clear from the text if there was any spatial rule (i.e. conduction times) or whether they were set randomly. The range corresponds well to an axonal arbor with 150 um radius in L2 of the mEC.

*This is now noted in the text at lines 819-821.*

*“No spatial structure was assigned to the network, and the conduction delay for each synapse was assigned from a uniform distribution ranging from 0.6 to 1.0 ms.’*

*The lack of a correlation between distance between somas and delay is shown below (unpublished data provided by Fernando Fernandez in the White lab).*

Reviewer Figure 5

8. In fact, the short range of connectivity of basket cells is a feature of the mEC which contrasts the wide axonal arbors of basket cells (500 - 1000 um) in the hippocampus/dentate gyrus. Please consider this difference and its impact between the models for the hippocampus and the mEC in the discussion.

*We added this paragraph to lines 663-669 of the Discussion:*

*“Another difference between brain regions is the short synaptic delays (between 0.6 -1.0 ms) observed in the mEC, which corresponds well to an axonal arbor with 250 μm radius in L2 of the mEC* (Martínez et al. 2017)*, assuming a floor of about 0.4 ms for the synaptic delay and a conduction velocity on the order (0.5 m/s). The short range of connectivity of basket cells is a feature of the mEC which contrasts with the wide axonal arbors of basket cells (500 - 1740 μm) in the hippocampus rat* (Sik et al. 1995) *and dentate gyrus* (Degro et al. 2022)*. Perhaps this feature contributes to highly localized synchrony involved in mEC grid cell assemblies* (Pastoll et al. 2013)*.”*

9. In Figure 4 legend A2 & 3 and B2 & 3 are not explained.

*This has been corrected.*

10. Text repeatedly refers to non-existing Figure 4C (e.g. line 210, 286, 319 and Fig 5 legend)

*References to Fig 4C (now Fig. 3) were corrected to refer to Fig. 3A3 or B3 as appropriate.*

11. I find it somewhat inconvenient that the panel labels in the figures are not aligned to the left of the panels, for easy orientation, but have variable positions and are merged with the titles (in some without any space between them, e.g. Figure 5BCD).

*Figure 5 has been split into Figures 4 and 5 at the request of Reviewer* 1, *but the offending labels have been corrected and aligned.*

12. There are some minor typos and punctuation errors (e.g. lines 103, 105, 219).

*Corrected.*

**Achuthan S**, **Canavier CC**. Phase-Resetting Curves Determine Synchronization, Phase Locking, and Clustering in Networks of Neural Oscillators. *J Neurosci* 29: 5218–5233, 2009.

**Belluscio MA**, **Mizuseki K**, **Schmidt R**, **Kempter R**, **Buzsaki G**. Cross-Frequency Phase-Phase Coupling between Theta and Gamma Oscillations in the Hippocampus. *J Neurosci* 32: 423–435, 2012.

**Bi H**, **Segneri M**, **di Volo M**, **Torcini A**. Coexistence of fast and slow gamma oscillations in one population of inhibitory spiking neurons. *Phys Rev Res* 2: 013042, 2020.

**Bragin A**, **Jandó G**, **Nádasdy Z**, **Hetke J**, **Wise K**, **Buzsáki G**. Gamma (40-100 Hz) oscillation in the hippocampus of the behaving rat. *J Neurosci* 15: 47–60, 1995.

**Butler JL**, **Hay YA**, **Paulsen O**. Comparison of three gamma oscillations in the mouse entorhinal-hippocampal system. *Eur J Neurosci* 48: 2795–2806, 2018.

**Butler JL**, **Mendonça PRF**, **Robinson HPC**, **Paulsen O**. Intrinsic Cornu Ammonis Area 1 Theta-Nested Gamma Oscillations Induced by Optogenetic Theta Frequency Stimulation. *J Neurosci Off J Soc Neurosci* 36: 4155–4169, 2016.

**Chrobak JJ**, **Buzsáki G**. High-frequency oscillations in the output networks of the hippocampal-entorhinal axis of the freely behaving rat. *J Neurosci Off J Soc Neurosci* 16: 3056–3066, 1996.

**Colgin LL**, **Denninger T**, **Fyhn M**, **Hafting T**, **Bonnevie T**, **Jensen O**, **Moser M-B**, **Moser EI**. Frequency of gamma oscillations routes flow of information in the hippocampus. *Nature* 462: 353–357, 2009.

**Coombes S**, **Byrne A**. Next Generation Neural Mass Models. In: *Nonlinear Dynamics in Computational Neuroscience*. Cham, Switzerland: Springer International Publishing, 2019, p. 1–16.

**Cunningham MO**, **Halliday DM**, **Davies CH**, **Traub RD**, **Buhl EH**, **Whittington MA**. Coexistence of gamma and high-frequency oscillations in rat medial entorhinal cortex in vitro. *J Physiol* 559: 347–353, 2004.

**Degro CE**, **Bolduan F**, **Vida I**, **Booker SA**. Interneuron diversity in the rat dentate gyrus: An unbiased in vitro classification. *Hippocampus* 32: 310–331, 2022.

**Deleuze C**, **Bhumbra GS**, **Pazienti A**, **Lourenço J**, **Mailhes C**, **Aguirre A**, **Beato M**, **Bacci A**. Strong preference for autaptic self-connectivity of neocortical PV interneurons facilitates their tuning to γ-oscillations. *PLOS Biol* 17: e3000419, 2019.

**Dumont G**, **Ermentrout GB**, **Gutkin B**. Macroscopic phase-resetting curves for spiking neural networks. *Phys Rev E* 96: 042311, 2017.

**Dumont G**, **Gutkin B**. Macroscopic phase resetting-curves determine oscillatory coherence and signal transfer in inter-coupled neural circuits. *PLoS Comput Biol* 15: e1007019, 2019.

**Fernandez FR**, **Via G**, **Canavier CC**, **White JA**. Kinetics and Connectivity Properties of Parvalbumin- and Somatostatin-Positive Inhibition in Layer 2/3 Medial Entorhinal Cortex. *eNeuro* 9: ENEURO.0441-21.2022, 2022.

**Geisler C**, **Brunel N**, **Wang X-J**. Contributions of intrinsic membrane dynamics to fast network oscillations with irregular neuronal discharges. *J Neurophysiol* 94: 4344–4361, 2005.

**Goldberg EM**, **Clark BD**, **Zagha E**, **Nahmani M**, **Erisir A**, **Rudy B**. K+ channels at the axon initial segment dampen near-threshold excitability of neocortical fast-spiking GABAergic interneurons. *Neuron* 58: 387–400, 2008.

**Gu Y**, **Servello D**, **Han Z**, **Lalchandani RR**, **Ding JB**, **Huang K**, **Gu C**. Balanced Activity between Kv3 and Nav Channels Determines Fast-Spiking in Mammalian Central Neurons. *iScience* 9: 120–137, 2018.

**Hodgkin AL**. The local electric changes associated with repetitive action in a non-medullated axon. *J Physiol* 107: 165–181, 1948.

**Izhikevich EM**. Resonate-and-fire neurons. *Neural Netw* 14: 883–894, 2001.

**Izhikevich EM**. *Dynamical Systems in Neuroscience*. MIT Press, 2007.

**Mann EO**, **Paulsen O**. Role of GABAergic inhibition in hippocampal network oscillations. *Trends Neurosci* 30: 343–349, 2007.

**Martínez JJ**, **Rahsepar B**, **White JA**. Anatomical and Electrophysiological Clustering of Superficial Medial Entorhinal Cortex Interneurons. *eNeuro* 4, 2017.

**Pastoll H**, **Solanka L**, **van Rossum MCW**, **Nolan MF**. Feedback inhibition enables θ-nested γ oscillations and grid firing fields. *Neuron* 77: 141–154, 2013.

**Pazó D**, **Montbrió E**. Low-Dimensional Dynamics of Populations of Pulse-Coupled Oscillators. *Phys Rev X* 4: 011009, 2014.

**Segneri M**, **Bi H**, **Olmi S**, **Torcini A**. Theta-Nested Gamma Oscillations in Next Generation Neural Mass Models. *Front Comput Neurosci* 14: 47, 2020.

**Sik A**, **Penttonen M**, **Ylinen A**, **Buzsáki G**. Hippocampal CA1 interneurons: an in vivo intracellular labeling study. *J Neurosci Off J Soc Neurosci* 15: 6651–6665, 1995.

**Taher H**, **Torcini A**, **Olmi S**. Exact neural mass model for synaptic-based working memory. *PLoS Comput Biol* 16: e1008533, 2020.

**Tikidji-Hamburyan RA**, **Leonik CA**, **Canavier CC**. Phase Response Theory Explains Cluster Formation in Sparsely but Strongly Connected Inhibitory Neural Networks and Effects of Jitter due to Sparse Connectivity. *J Neurophysiol* , 2019. doi:10.1152/jn.00728.2018.

**White JA**, **Chow CC**, **Ritt J**, **Soto-Treviño C**, **Kopell N**. Synchronization and oscillatory dynamics in heterogeneous, mutually inhibited neurons. *J Comput Neurosci* 5: 5–16, 1998.

**Williams B**, **Fernandez FR**, **Canavier CC**, **White JA**. Fast Spiking Interneurons Generate Gamma Oscillations in the Medial Entorhinal Cortex without Excitatory Input. *Soc Neurosci Meet Plan* 132.02, 2021.

**Winfree AT**. Biological rhythms and the behavior of populations of coupled oscillators. *J Theor Biol* 16: 15–42, 1967.

**Yamamoto J**, **Tonegawa S**. Direct Medial Entorhinal Cortex Input to Hippocampal CA1 Is Crucial for Extended Quiet Awake Replay. *Neuron* 96: 217-227.e4, 2017.
